# Genetic and environment effects on structural neuroimaging endophenotype for bipolar disorder: a novel molecular approach

**DOI:** 10.1038/s41398-022-01892-3

**Published:** 2022-04-04

**Authors:** Bo Hu, Jungwon Cha, Janice M. Fullerton, Sonia Hesam-Shariati, Kunio Nakamura, John I. Nurnberger, Amit Anand

**Affiliations:** 1 Department of Quantitative Health Sciences, Cleveland Clinic, Cleveland, OH USA; 2 Department of Psychiatry, Brigham and Women’s Hospital, Harvard Medical School, Boston, MA USA; 3 Neuroscience Research Australia, Sydney, NSW Australia; 4 School of Medical Sciences, University of New South Wales, Sydney, NSW Australia; 5 Department of Biomedical Engineering, Cleveland Clinic, Cleveland, OH USA; 6 Departments of Psychiatry and Medical and Molecular Genetics, Stark Neurosciences Research Institute, Indiana University School of Medicine, Indianapolis, IN USA

**Keywords:** Bipolar disorder, Genetics

## Abstract

We investigated gene–environment effects on structural brain endophenotype in bipolar disorder (BD) using a novel method of combining polygenic risk scores with epigenetic signatures since traditional methods of examining the family history and trauma effects have significant limitations. The study enrolled 119 subjects, including 55 BD spectrum (BDS) subjects diagnosed with BD or major depressive disorder (MDD) with subthreshold BD symptoms and 64 non-BDS subjects comprising 32 MDD subjects without BD symptoms and 32 healthy subjects. The blood samples underwent genome-wide genotyping and methylation quantification. We derived polygenic risk score (PRS) and methylation profile score (MPS) as weighted summations of risk single nucleotide polymorphisms and methylation probes, respectively, which were considered as molecular measures of genetic and environmental risks for BD. Linear regression was used to relate PRS, MPS, and their interaction to 44 brain structure measures quantified from magnetic resonance imaging (MRI) on 47 BDS subjects, and the results were compared with those based on family history and childhood trauma. After multiplicity corrections using false discovery rate (FDR), MPS was found to be negatively associated with the volume of the medial geniculate thalamus (FDR = 0.059, partial *R*^2^ = 0.208). Family history, trauma scale, and PRS were not associated with any brain measures. PRS and MPS show significant interactions on whole putamen (FDR = 0.09, partial *R*^2^ = 0.337). No significant gene–environment interactions were identified for the family history and trauma scale. PRS and MPS generally explained greater proportions of variances of the brain measures (range of partial *R*^2^ = [0.008, 0.337]) than the clinical risk factors (range = [0.004, 0.228]).

## Introduction

Gene–environment interaction is recognized to be important in the development and manifestation of complex illnesses such as major psychiatric disorders. Bipolar disorder (BD) is a major mental illness which is frequently familial, suggesting genetic factors contribute to disease risk. Also, evidence from several studies, including studies by the current investigators [1–4], indicate that environmental factors and gene–environment interaction may play a role in the development and psychopathology of bipolar disorder.

### Genetics of bipolar disorder and familial transmission

BD is one of the most heritable psychiatric illnesses with 40–70% concordance in monozygotic twins, and a population incidence of 1–4%, but incidence increases to 10–50% if both parents have BD, or 10–25% if one parent or sibling has BD [5, 6]. Therefore, BD has been the subject of intense gene discovery research for past decades, and a number of genes and single nucleotide polymorphisms (SNPs) have been identified as increasing disease risk, including the CACNAC1 gene [7, 8]. However, the contribution of these individual SNPs has been modest, at the rate of less than 2%, and heritability estimates from genotype data (which currently model only additive genetic effects from SNPs, *h*^2^ = ~23%) [7] are well below that derived from family, twin and epidemiology studies. Therefore, the current view is that bipolar illness is a complex multifactorial polygenic disorder, which may involve numerous genetic factors interacting with other genes and/or environmental factors. Despite this realization, formal gene and environmental interaction studies have been scarce, and the emphasis in the field has mainly been on increasing power for genetic studies of bipolar disorder.

### Environmental effects and BD

We previously reported that childhood trauma is associated with the earlier age of onset of bipolar illness and that calcium channel pathway genes may interact with childhood trauma to influence the age of onset of bipolar illness [1]. We and others have also reported the effect of childhood abuse and substance abuse on the emergence of bipolar illness [4, 9]. A number of studies have documented the role of childhood trauma in depression and BD [4, 10, 11].

### Familial and sporadic research design

The familial-sporadic design has been proposed as one method to differentiate between genetic and environmental factors [12]. This design is based upon the assumption that patients with one or more relatives with the same illness, particularly first-degree relatives, are more likely to have a genetic basis to their illness, while patients with no such family history (sporadic) are likely to have environmental determinants of their disorder. One major limitation of this method is that familiality is used as a proxy for the underlying genetic basis of the disorder. Familiality can be inaccurately estimated for a variety of reasons such as a small family size (providing a reduced opportunity for trait transmission), poor family communication of health status, or the age at which relatives are assessed (when illness symptoms may not yet have emerged). Furthermore, familiality confounds both genetic and environmental factors, as first-degree relatives are likely to be exposed to a similar environment, and children of BD probands may experience more environmental stress as a result of parental illness thereby confounding ‘familiality’ as a proxy for genetic vulnerability [12]. Therefore, a more objective measure of genetic vulnerability for the illness is needed.

### Polygenic risk score (PRS)

The polygenic risk score is one objective indicator of the genetic basis of a disorder [13]. Genome-wide association studies (GWAS) have recently identified 30 genome-wide significant susceptibility loci for BD [7, 8]. Common SNPs in aggregate appear to explain 25–40% of the liability to BD [14], and current studies suggest that rare variants and gene–gene interactions explain substantial fractions of the remaining genetic contribution to BD risk [15–17]. PRS is a cumulative measure of genomic risk, as calculated by the sum of trait-associated alleles across many genetic loci, typically weighted by effect sizes estimated from a GWAS. PRS can be used to establish the presence of a genetic signal in an independent target sample, to index the underlying genetic burden of a trait, and as a biomarker for a phenotype. In this respect, it can be argued to be a more objective and specific genetic background measure than “familiality”, but also not so granular as a measure of inheritance of a single gene variant or SNP. For the purpose of the current study, which aimed to tease out genetic versus environmental effects and their interaction in the manifestation of bipolar illness, the PRS approach is highly suitable as it has been derived from the largest available GWAS, which is applicable to a small number of densely phenotyped subjects.

### Epigenetic risk

DNA methylation refers to alterations in the genome that do not alter nucleotide sequence, and typically occur via the addition of a methyl group to the carbon-5 position of a cytosine residue (CpG). Methylation of CpG sites which occur within transcript regulatory regions can lead to changes in gene expression and are often attributed to environmental effects. The growing recognition that exposure to environmental stressors can be embedded in the genome via epigenetic modifications, both during the early stages of development and in adulthood, has led to increasing interest in characterizing epigenetic signatures as a mechanism for how exposure to stressors may have long-term effects which are associated with the development of psychiatric disorders. While some large-scale (*n* > 500) epigenome-wide association studies (EWAS) are available for schizophrenia and other psychiatric disorders [18–20], only small cohort studies thus far exist for BD in the literature. Therefore, an epigenetic risk score, as a weighted summation of risk CpG sites, with the CpG sites and their weights identified from a well-powered independent EWAS study, is not available for BD. However, various alternatives have been proposed to develop internal weights for CpG sites within an experimental sample [21]. To avoid the issue of overfitting, regularization methods such as LASSO or some dimension reduction methods can be applied [22–24].

### Gene and environmental interaction in BD

The measurement and independent analysis of genetic and environmental factors does not provide information regarding combinatorial effects and how genetic and environmental factors may interact to cause illness. As our primary comparator groups both have bipolar symptoms, such an investigation needs to be conducted with an available endophenotype which is different in familial and sporadic illness. Variability in brain morphology provides such an endophenotype with which the underlying genetic, environmental, and interactional effects can be studied in the context of BD.

### Structural imaging endophenotype in BD

In the last few decades, a number of corticolimbic structural abnormalities have been reported in depression and BD. For example, structural changes in hippocampal, amygdala, striatum, and frontal cortical regions have been reported [25, 26]. Moreover, structural abnormalities have also been reported in relation to environmental effects, in particular childhood and recent trauma [27]. Therefore, regional brain structural features can be used as an endophenotype for genetic and environmental effects and their interactions.

We hypothesized that besides family history, PRS will provide a more reliable molecular measure of genetic risk for bipolar illness. Similarly, we hypothesized that a summary epigenetic methylation profile score (MPS) will provide a reliable measure of environmental effects. The gene–environment interaction effects were further tested by relating molecular and clinical measures, respectively, to brain structural endophenotypes.

## Materials and methods

### Subjects

The study was approved by Cleveland Clinic IRB. The BD spectrum group (BDS) included subjects with bipolar disorder (BD) or subthreshold BD, who were recruited from the outpatient psychiatry clinic at the Cleveland Clinic by advertisement [28]. The BDS subjects were 15–30 years old, and were not on antidepressants and mood stabilizers at least in the past 2 weeks, and not on fluoxetine in the past 5 weeks before enrollment. We also enrolled a closely matched group of depressed subjects with no subthreshold BD symptoms or family history of BD, as well as healthy control subjects. The non-BDS subjects were included to derive a methylation risk score but were not included in the association analyses for brain structure measures. Detailed inclusion and exclusion criteria are presented in the supplementary materials. A peripheral blood sample was collected from each subject for genetic and epigenetic profiling, as detailed below.

### Familiality, genetic, and environmental Factors

*Family history* of bipolar disorder was defined from self-report as having either a first- or second-degree relative with BD. *Early-life trauma* was documented using the Childhood Life Events Scale (CLES) [1], an 11-point scale that details various traumatic events that may have happened between ages 3 and 12. The final score is the number of accumulated traumas during childhood. *Life-time substance abuse history* (including alcohol and drug abuse) was obtained from the Mini International Neuropsychiatric Interview [29]. Smoking history was obtained by psychiatric interview.

#### Genotype and polygenic risk score (PRS)

The study subjects were genotyped from blood-derived DNA using the Illumina Infinium PsychChip (Illumina Inc, San Diego, CA). Quality control (QC) procedures included filtering SNPs based on GenCall score (GC <0.2), call rate (<95%), minor allele frequency (<1%), and Hardy–Weinberg equilibrium (HWE *p* < 1e-6) using PLINK v1.9 [30]. Subjects with a low genotyping rate (<95%) and sex inconsistencies (one subject) were excluded. Spurious relatedness between participants was examined by identity-by-descent (IBD) analysis of independent SNP markers, and no significant relationships were detected ($\hat{\pi}$ < 0.14, indicating only a few third-degree relatives). The genotype data passing QC were then imputed using the Michigan Imputation Server [31]. The allele frequency correlation between reference and sample data was *r*^2^ = 0.966.

After imputation, the genotype data underwent additional QC (HWE *p* < 1e-20, indels, and duplicated SNPs removed) and ~10.9 million SNPs with imputation *R*^2^ > 0.3, and MAF >0.01 were retained and used to calculate the polygenic risk score for BD, following the method described by the International Schizophrenia Consortium using PRSice v1.25 [32, 33]. The risk SNPs and their respective effect size weights were obtained from the GWAS for BD performed by the Psychiatric Genomics Consortium Bipolar Disorder Working Group [7]. For each subject, the PRS was calculated as a summation of the number of risk alleles multiplied by the corresponding weights for the set of SNPs selected at a maximally informative *p* value threshold (*p*_T_). We first computed the polygenic risk scores at *p*_T_’s ranging from 0.01 to 0.5 with an increment of 0.01. Then a series of logistic regression models were fitted to relate each PRS to family-history positive and family-history negative groups. The PRS at *p*_T_ = 0.07 led to the most significant difference between the groups (Supp. Fig. 1a), which was employed in the downstream association analyses.

#### Methylation and methylation profile score (MPS)

DNA Methylation quantification from the peripheral blood samples employed the Illumina MethylationEPIC BeadChip, as per the manufacturer’s standard protocol. Methylation data output from GenomeStudio were further analyzed using the R *minfi* package [34]. Quality control was performed to remove CpG probes with (1) a low detection rate at *P* < 0.01; (2) bead count <3 in at least 5% of the samples; and (3) an SNP at either the CpG interrogation or the single nucleotide extension site. Subject-level QC included (1) outlier detection using the first two principal components and (2) confirming subject-reported gender information with methylation-derived gender estimates. Finally, the data were normalized using the BMIQ method and were further logit-transformed.

Unlike GWAS, there are no existing large-scale epigenome-wide association studies for BD. We applied the feature selection approach of LASSO to derive a methylation profile risk score (MPS) [21, 23]. More specifically, a logistic regression model was fit to relate methylation probes to the binary outcome of BDS, and LASSO was performed using the *glmnet* package in R (cran.r-project.org) for probe selection. The MPS was then computed as the weighted summation of the selected risk probes (i.e., $\mathop {\sum }\nolimits_{i = 1}^m w_ix_i$), where $w_i$ represents the weight for the selected probe $x_i$ and *m* is the number of probes selected.

### MRI acquisition and imaging analysis

MRI Scans were performed using a Siemens 3 T Tim Trio. After a short scout imaging scan to survey head position and center the field of view (FOV), a high-resolution 3D magnetization prepared rapid gradient echo (MPRAGE) scan was performed and used for structural analyses. This high-resolution anatomical volume comprised of 160 sagittal slices and had 1.0 mm × 1.0 mm × 1.2 mm voxel dimensions. The scans were acquired with a repetition time (TR) of 2300 ms, echo time (TE) of 2.91 ms, flip angle of 9°, and FoV of 240 × 256.

T1w MPRAGE was preprocessed using iterative N3 intensity correction [35] and standard space (International Consortium for Brain Mapping [36]) registration using a hierarchical approach [37]. Inter-session scans were co-registered using MINC (Medical Imaging NetCDF) toolkit (V2 1.9.16) [38]. Three types of brain structural measures were calculated: gray and white matter fractions, cortical thicknesses, and volumes of subcortical structures.

Whole-brain fraction (WBF) was calculated as the ratio of brain parenchymal volume and outer brain contour volume, and the result was given in an arbitrary fractional unit. Gray matter fraction (GMF) and white matter fraction (WMF) were calculated using Statistical Parametric Mapping (SPM) version 12 to segment MPRAGE image into gray matter, white matter, and cerebrospinal fluid and further combined with FSL’s (FMRIB Software Library) FIRST segmentation masks [39]. The volumes of gray matter and white matter were divided by the intracranial volume, which was derived from the standard ICBM atlas to result in GMF and WMF in arbitrary units.

Cortical thickness was measured using a cortical longitudinal atrophy detection algorithm (CLADA) [40], which was developed internally. FSL’s FIRST (FSL version 5.0.9) [41] was used to calculate the normalized volumes of accumbens, amygdala, caudate, hippocampus, pallidum, putamen, and thalamus on each hemisphere. Thalamic subnuclei were segmented using an atlas-based method where T1w MPRAGE is nonlinearly registered using ANTS [42] to transform the labels: anterior, centromedian, habenula, lateral, lateral geniculate, medial, medial geniculate, mediodorsal, and pulvinar nuclei of the thalamus. The entire list of brain structural measures analyzed in the current paper are provided in Supplementary Table 1.

### Statistical analysis

Subjects’ demographic, clinical, and molecular characteristics were summarized with appropriate descriptive statistics. Dichotomous variables were compared using the chi-squared test, while continuous variables, including age, trauma scale, PRS, and MPS, were compared using the Wilcoxon rank-sum test.

Examination of the genetic and environmental effects on brain structure measures was restricted to BDS subjects. The univariate relationship was evaluated using the Spearman correlation coefficient. To further adjust for covariates of age, gender, self-reported race (Caucasian or African American), and smoking status, linear regression models were applied. For example, for the relationship between gray matter fraction (GMF) and PRS, the model has the form

$${{{\mathrm{GMF}}\sim {\mathrm{PRS}} + {\mathrm{age}} + {\mathrm{age}}}}^{{{\mathrm{2}}}}\,+\,{\mathrm{gender}} + {\mathrm{race}} + {\mathrm{smoking}}\;{\mathrm{status}},$$

where PRS can be substituted with a family history of BD, CLES, or MPS, and the dependent variable of GMF can be substituted with other brain structural measures. All structural measures were standardized before modeling. Note that the term of age^2^ is included since it was found to be significantly related to the brain features in the multivariate analysis (*p* = 0.023 from Pillai’s trace test).

Genetic and environmental interactions were also examined using linear regression. More specifically, for the pair of molecular measures (i.e., PRS and MPS), we fitted the following model:

GMF ~ PRS + MPS + PRS:MPS + age + age^2^ + gender + race + smoking status, where the term PRS:MPS represents the gene–environment interaction. The same model was fitted for the pair of clinical measures of BD risks (i.e., family history of BD and CLES). For each model, partial *R*^2^ statistic was calculated to quantify the proportion of variance in the brain measure explained by the genetic and environmental factors adjusting for the covariates. The partial *R*^2^ was calculated as one minus the ratio of the sum of squared residuals (SSR) of the model with genetic and environmental factors and the SSR of the model with only covariates [43, 44]. The calculations of partial *R*^2^ were performed using the *sensemakr* package in R. Bootstrap with 2000 runs was used to compare partial *R*^2^ statistics from the model with clinical measures to that with molecular measures.

False discovery rates were obtained using the *q*-value approach and statistical significance was established at FDR <0.1. Sensitivity analyses were performed by replacing race with ancestry estimated from the genotype data. We also performed a subgroup analysis for Caucasians. All statistical analyses were conducted using R-studio (Boston, MA).

## Results

For the BDS subjects, the median age was 23 (inter-quartile range = [20, 26]), 72.7% were female and 74.5% were Caucasian (Table 1). For the non-BDS subjects, the median age was 25 (inter-quartile range = [22, 28]), 59.4% were female and 82.8% were Caucasian. Three (5.5%) BDS subjects had a history of alcohol abuse and five (9.1%) had a history of drug abuse. Non-BDS subjects were free of substance abuse.

**Table 1:** Demographics and illness characteristics of study subjects.

		Non-BDS (*N* = 64)		BDS (*N* = 55)		*p*
Age		25	[22, 28]^a^	23	[20, 26]	0.015
Gender	Female	38	59.4%	40	72.7%	0.182
	Male	26	40.6%	15	27.3%	
Race	African American	5	7.8%	14	25.5%	0.004
	Asian	6	9.4%	0	0.0%	
	Caucasian	53	82.8%	41	74.5%	
Family history of BD		3	4.7%	25	45.5%	<0.001
Childhood trauma		45	70.3%	51	94.4%	0.002
Recent trauma		49	80.3%	44	83.0%	0.899
History of alcohol abuse		1	1.6%	3	5.5%	0.506
History of drug abuse		0	0.0%	5	9.1%	0.045
Smoking	No	38	66.7%	28	59.6%	0.459
	Yes	10	17.5%	13	27.7%	
	Unknown	9	15.8%	6	12.8%	
CLES^b^		3.67	[0, 5]	4.875	[3.575, 5.5]	0.011
PRS		−0.0147	[−0.0148, −0.0145]	−0.0146	[−0.0147, −0.0125]	0.048
MPS		−0.873	[−1.229, −0.574]	0.635	[0.456, 0.892]	<0.001

^a^Median [IQR] presented for continuous variables and *N* (%) for categorical variables.

^b^CLES is missing for one non-BDS and three BDS subjects.

45.5% of the BDS subjects had a family history of BD while only three (4.7%) non-BDS subjects had a positive family history of BD (*p* < 0.001). The PRS was higher in the BDS subjects (*p* = 0.048). 94.4% of the BDS subjects reported childhood traumas, which was significantly higher than 70.3% in the non-BDS group (*p* = 0.002). The Childhood Life Events Scale (CLES) was significantly higher in the BDS group (median scale: 4.875 vs 3.67, *p* = 0.011). No difference was found between BDS and non-BDS groups in relation to the experience of recent traumas.

The MPS of the BDS subjects was also significantly higher (*p* < 0.001). The calculation of MPS was based on 36 methylation probes selected by LASSO (Supp. Table 2). One of the probes, cg00111893 on gene ZNF259P1 (chromosome 6), was found to have significant me-QTL with thirteen SNPs (Supp. Table 3). However, these SNP-CpG pairs were not found in the existing me-QTL database [45].

### Genetic and environmental effects on structural brain measures

Eight BDS subjects did not undergo MRI scans and thus had no brain structure measures. PRS was found to be not significantly associated with any brain structural measures at FDR <0.1. Before multiplicity corrections, exploratory analyses showed that it was associated with the volumes of left accumbens (*r* = −0.149; ${\upbeta}$ = −1.056, *p* = 0.031, partial *R*^2^ = 0.114; Table 2) and right putamen (*r* = −0.113; ${\upbeta}$ = −1.268, *p* = 0.013; partial *R*^2^ = 0.149). Family history was not associated with any structure features (all nominal *p* values >0.05).

**Table 2:** Significant relationship between genetic or environmental factors and brain structure measures (nominal *p* < 0.05).

		Unadjusted analysis	Adjusted analysis*		
Factor	Brain measure	Spearman correlation (*r*)	*Beta*	*p*	Partial *R*^2^
PRS	Left accumbens	−0.149	−1.056	0.031	0.114
	Right putamen	−0.113	−1.268	0.013	0.149
MPS	Left accumbens	0.155	0.330	0.044	0.100
	Claustrum	−0.307	−0.385	0.022	0.127
	Medial geniculate thalamus	−0.142	−0.472	0.003^	0.208
	MTT thalamus	−0.235	−0.372	0.019	0.134
	Pulvinar thalamus	−0.248	−0.350	0.040	0.104

^*^Models include age, age^2^, gender, race, and smoking status as covariates.

^FDR < 0.1.

After multiplicity corrections, MPS was found to be significantly associated with the volume of medial geniculate thalamus (*r* = −0.142; ${\upbeta}$ = −0.472, *p* = 0.003, FDR = 0.059, partial *R*^2^ = 0.208). Based on the nominal *p* values <0.05, MPS was also associated with the volumes of left accumbens, claustrum, MTT thalamus, and pulvinar thalamus. CLES was not significantly correlated with any features (all nominal *p* values >0.05).

Since the genetic ancestry derived from genotype data and self-reported race were highly consistent (Supp. Fig. 1b), similar results were obtained by controlling for genetic ancestry instead of the self-reported race (results not shown). For the subgroup analysis of Caucasian subjects, the effects of the gene–environment factors were generally on the same scale as their effects on all subjects (Supp. Fig. 2), though as expected, no significant findings were identified pertaining to the smaller sample size.

### Gene–environment Interactions

**Fig. 1:**
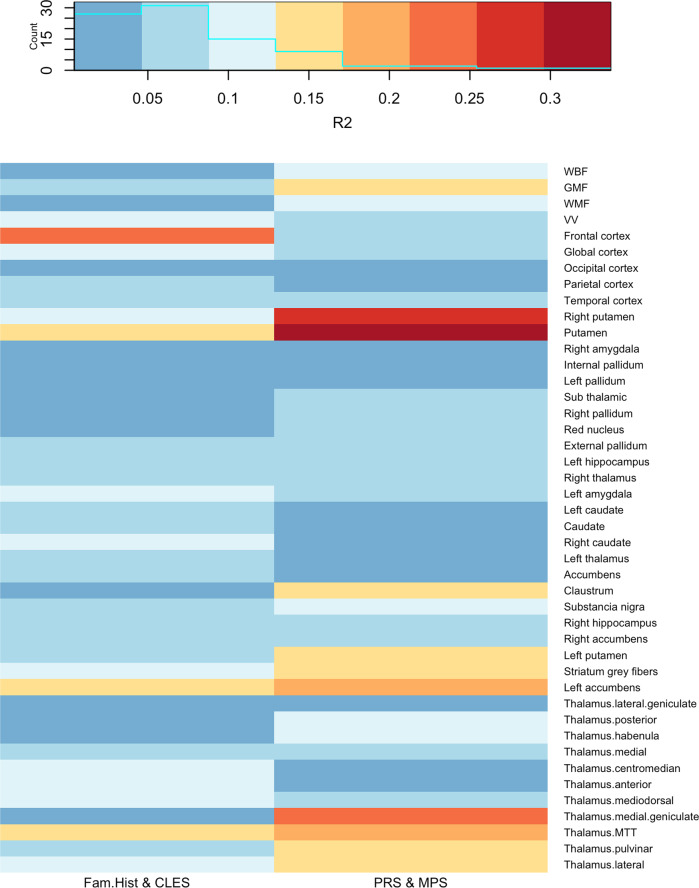
Partial *R*^2^ statistics for the interaction models relating clinical or molecular gene–environmental factors (i.e., family history and CLES or PRS and MPS) to brain structure measures.

Figure 1 shows that for 29 (66%) of the 44 brain measures examined, the models including PRS and MPS had higher partial *R*^2^ statistics than the models including family history and CLES (mean [range]: 0.089 [0.008, 0.337] vs. 0.070 [0.004, 0.228]). More specifically, the partial *R*^2^ statistics for right pallidum and subthalamic nucleus were significantly higher (*p* < 0.05) in the models with PRS and MPS.

At FDR <0.1, PRS and MPS had a significant gene–environment interaction on whole putamen (*p* = 0.002, FDR = 0.09, partial *R*^2^ = 0.337; Table 3). Based on the nominal *p* values, PRS and MPS also had significant interactions on both hemispheric putamen volumes: right putamen (*p* = 0.024, partial *R*^2^ = 0.285) and left putamen (*p* = 0.050, partial *R*^2^ = 0.163). Their interaction was also significant on gray matter fraction (*p* = 0.011, partial *R*^2^ = 0.167). Based on the nominal *p* values, family history and childhood trauma had significant interactions on frontal cortical thickness (*p* = 0.003, partial *R*^2^ = 0.228), global cortex thickness (*p* = 0.036, partial *R*^2^ = 0.124), right putamen (*p* = 0.049, partial *R*^2^ = 0.119) and whole putamen (*p* = 0.035, partial *R*^2^ = 0.142).

**Table 3:** Brain structure features with significant gene–environment interactions from (A) family history and CLES and (B) PRS and MPS, respectively.

A	Interaction of family history and CLES			
	***Beta***	***SE***	***p***	***Partial R***^***2***^
Frontal cortex thickness	−0.736	0.231	0.003	0.228
Global cortex thickness	−0.568	0.261	0.036	0.124
Right putamen	0.627	0.308	0.049	0.119
Putamen	0.756	0.344	0.035	0.142
**B**	**Interaction of PRS and MPS**			
	***Beta***	***SE***	***p***	***Partial R***^***2***^
Right putamen	−0.338	0.144	0.024	0.285
Left putamen	−0.32	0.158	0.050	0.163
Putamen	−0.466	0.14	0.002^^^	0.337
GMF	0.364	0.135	0.011	0.167

^FDR <0.1.

## Discussion

In this study, using clinical as well as objective molecular measures, we investigated gene–environment effects on the brain structural endophenotype in BDS.

A number of studies have shown the abnormalities of the thalamus and thalamic subregions in BD [46–49]. The thalamus acts as a relay station for sensory information. Excessive or intensive sensory stimulation associated with trauma or other risk factors, particularly in the developmental stages, could lead to structural and functional changes in the thalamus. Our results indicate that after corrections for multiplicity, MPS was significantly inversely correlated with the volume of the median geniculate nucleus of the thalamus. The median geniculate nucleus relays auditory sensory information to the auditory cortex and is therefore integrally involved in the effect of the environment. Furthermore, it has been shown to have direct connections with the amygdala and the median geniculate-amygdala circuit has been implicated in the encoding of auditory fear memories and generalization of fear response to unconditioned stimuli [50]. Keeping these functional for median geniculate thalamic nucleus in mind, it is interesting that MPS was shown to be inversely correlated with its volume. Trends were also seen for MPS to be correlated negatively with a volume of the pulvinar (involved in visual information processing) and the mammillothalamic tract (involved in emotional information processing). Together these findings suggest that the effect of environmental trauma on the brain can be studied using the correlation of methylation signatures with brain structures.

Regarding gene–environment effects, a significant interaction between PRS and MPS was found for the volume of putamen bilaterally. Family history and childhood trauma score interaction also showed a trend-level interaction for putamen volumes along with frontal cortex thickness. The putamen is a major component of the dorsal striatum along with the caudate nucleus and both are rich in dopamine. Putamen volume has been shown to have 77% heritability [51] and is associated with genes implicated in a number of neuropsychiatric disorders [52]. The putamen is involved in motor movement and many studies have been shown its abnormal structure and function in BD [53–55]. Importantly, putamen volume and function changes have been shown in post-traumatic stress disorder (PTSD). Recent studies have shown a negative correlation between childhood trauma and putamen volume [56, 57]. In addition, fMRI studies have reported increased putamen activation to unconditioned stimulus and decreased putamen-amygdala connectivity in PTSD subjects [58–60]. The results of our study show that abnormal putamen structure and function could be used as biomarkers of gene–environment interaction. The greater magnitude of interaction seen with MPS and PGS compared to a childhood trauma history and family history suggest that molecular measures may be more sensitive in elucidating the effect of genetics and trauma on putamen structure and function.

This proof-of-concept study showed that PRS and MPS can be used to study the main effects of genes and environment, as well as their interaction on BD. This approach, based on objective laboratory measures rather than clinical and historical measures such as family history and trauma effects, is less prone to subjective distortion. Therefore, it can be adopted in future larger studies to uncover genetic and environmental effects in BD or other psychiatric disorders or extended into other medical illnesses.

Limitations of the study included that the behavioral data collected were retrospective in nature, in regard to recall of childhood traumatic events. Prospective studies will be illuminating in offspring at high risk for bipolar disorder because of family history. Secondly, our study had a small sample size and thus had limited statistical power for identifying and validating individual methylation probes associated with BD, or defining/disambiguating those associated with confounding demographic features. Furthermore, due to the small sample size, we were only able to identify significant relationships between MPS and medial geniculate thalamus after corrections for multiplicity. Other findings based on the nominal *p* values need to be considered as exploratory. Thus a large-scale study is highly desired to overcome these limitations. This may include the development of a large epigenome-wide reference data set for BD, with which a validated epigenetic risk score, similar to PRS, can be calculated. This approach has already been followed for other psychiatric disorders such as schizophrenia [18, 19], depression [61], and other medical disorders [21, 62]. A large cohort can also lead to more accurate race-specific data since both MPS and PRS may index molecular signatures which are variable with ancestry.

In conclusion, the results of our study provide promising preliminary data identifying environmental factors that may interact with a genetic vulnerability to the bipolar disorder spectrum, thereby leading to brain structural changes. Furthermore, this study provides a novel approach to study gene–environmental effects in psychiatric disorders and other illnesses, particularly where large discovery datasets are unavailable.

## Supplementary information

Supplementary Materials

Supplementary Figure 1

Supplementary Figure 2
