# Supplementary material for: Genetic and environment effects on structural neuroimaging endophenotype for bipolar disorder: a novel molecular approach
Source: Transl Psychiatry. 2022 Apr 4;12:137. doi: 10.1038/s41398-022-01892-3 (PMC8980067; doi:10.1038/s41398-022-01892-3)
Supplement: Supplementary file 1 — Supplementary Materials [file 41398_2022_1892_MOESM1_ESM.docx]

**SUPPLEMENTARY MATERIALS**

**Inclusion criteria for BDS subjects include**: 1) ages 15 - 30 years (inclusive) and able to give voluntary informed consent (consent taken from both parents and child for children under the age of 18); 2) satisfy criteria for Diagnostic and Statistical Manual 4th edition Text Revision (DSM-IV-TR) depressive episode-current; 3) 17-item Hamilton Depression Rating Scale > 15 but < 25; 4) Young Mania Rating Scale score < 10; 5) able to be managed as outpatients during the study as ascertained by the following: i. Clinical Global Severity Scale < 5 (moderately ill); ii. no significant suicidal or homicidal ideation or severe disability.

BD subjects further met Diagnostic and Statistical Manual 4th edition (DSM-IV-TR) criteria for Bipolar I or II disorder or Bipolar NOS. For Bipolar NOS we used a conservative criterion: euphoric mood with at least 2 mania symptoms or increased irritability with 3 mania symptoms if only the latter was present, as well as if more mania symptoms were present then duration was less than 4 days.

**Exclusion criteria for BDS subjects:** include: 1) meeting DSM-IV criteria for schizophrenia, schizophreniform disorder, schizoaffective disorder, mental retardation, pervasive developmental disorder; 2) history of receiving electroconvulsive therapy in the past 1 year; 3) use of neuroleptics, mood stabilizers or benzodiazepines in the past 2 weeks; 4) use of antidepressants in the past 2 weeks; 5) if on fluoxetine in the past, then should not have been on this medication for 5 weeks; 6) acutely suicidal or homicidal or requiring inpatient treatment; 7) meeting DSM-TR criteria for other substance/alcohol dependence within the past 6 months or abuse in the past 3 months, excluding caffeine or nicotine. The criteria were evaluated by interview and urinary toxicology screening; 8) use of alcohol in the past 1 week; 9) no serious acute or chronic medical or neurological illness, including previously known HIV-positive status (due to possible CNS involvement) as assessed by history, physical examination and laboratory examination including EKG, CBC and blood chemistry; 10) current pregnancy or breastfeeding; 11) metallic implants or another contraindication to MRI.

**Inclusion criteria for healthy subjects:** 1) ages 15 - 30 years (inclusive) and able to give voluntary informed consent (consent taken from both parents and child for children under the age of 18); 2) no current or past history of psychiatric illness or substance abuse or dependence; 3) no current or past history of psychiatric illness or substance abuse or dependence in a first-degree relative. Exclusion criteria for healthy subjects were the same as that for patients regarding pregnancy, contraindications to MRI, taking any psychotropic medications, any significant medical or neurological illness and use of alcohol in the past 1 week.

**Supplementary Figure 1.** (a) Comparison of polygenetic risk scores computed at different thresholds between BDS and non-BDS subjects, with significant and maximum distinction at *p*_T_=0.07; (b) genetic ancestry estimated from SNP genotyping data by self-reported race

| (a)  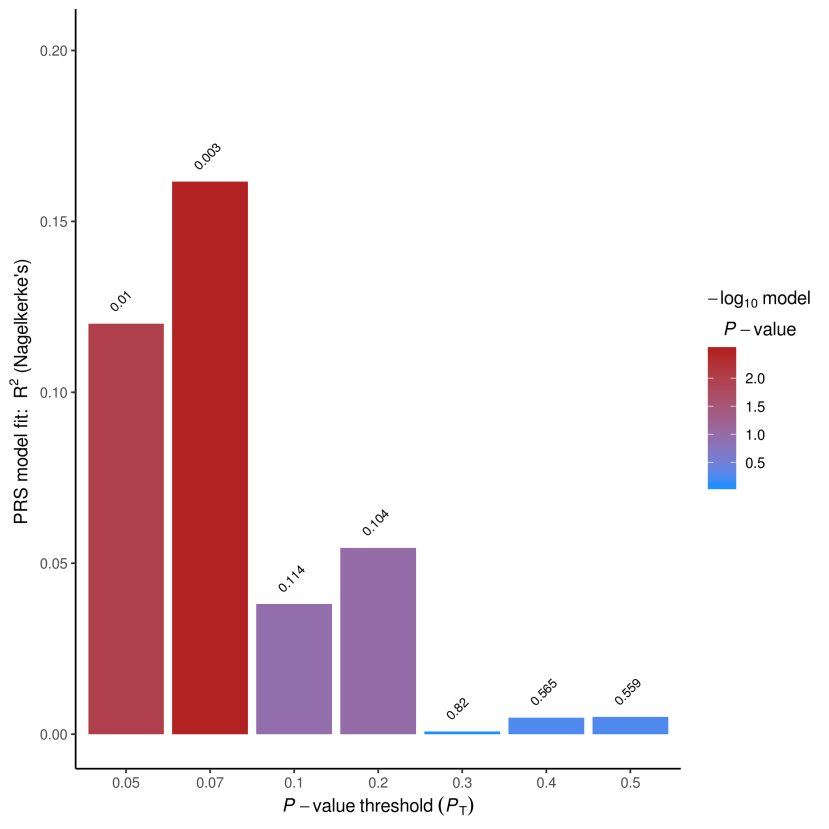 | (b)  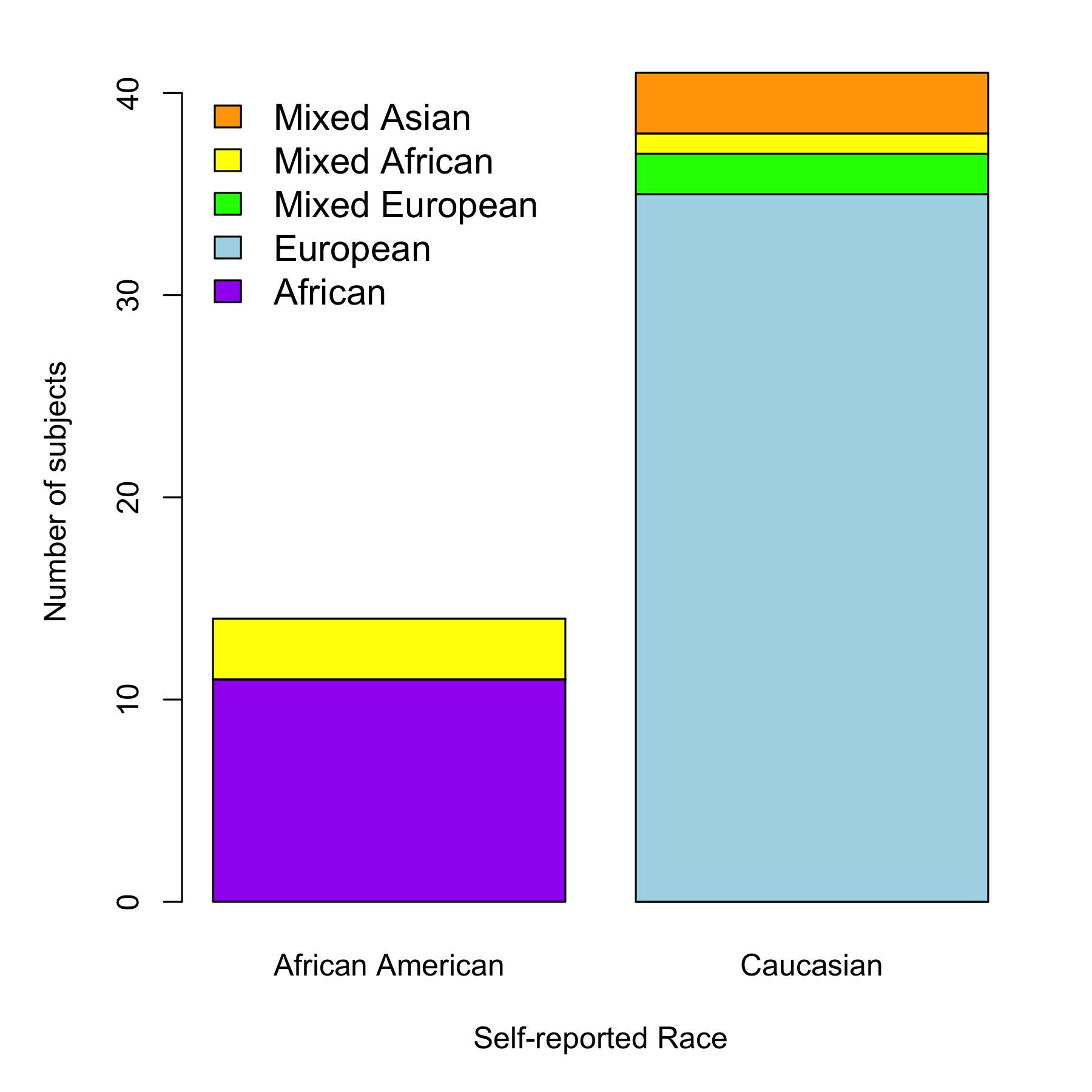 |
| --- | --- |

**Supplementary Figure 2**. Comparison of effects of individual genetic or environmental factors on brain measures for all subjects and those for Caucasians. Each dot represents the effect on one brain measure.

**
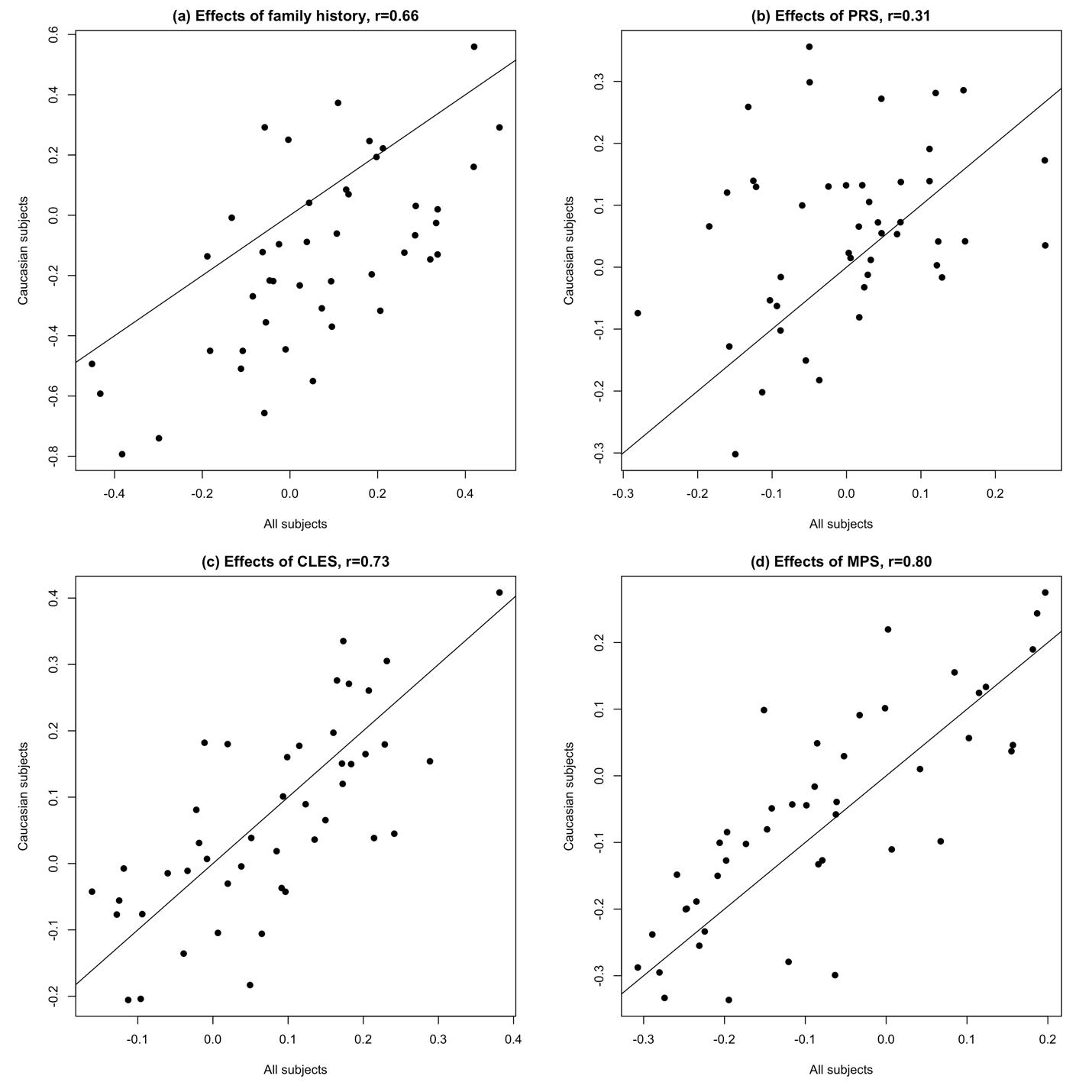
**

**Supplementary Table 1**. Brain Structure Measures Obtained from MRI Neuroimaging data

| Whole brain fraction (WBF) | Red nucleus volume |
| --- | --- |
| Gray matter fraction (GMF) | Striatum grey fibers volume |
| White matter fraction (WMF) | Sub thalamic volume |
| Ventricle volume (VV) | Substancia nigra volume |
| Frontal cortex thickness | Anterior thalamus volume |
| Global cortex thickness | Centromedian thalamus volume |
| Occipital cortex thickness | Habenula thalamus volume |
| Parietal cortex thickness | Lateral thalamus volume |
| Temporal cortex thickness | Lateral-geniculate thalamus volume |
| Accumbens volume (left and right)* | Medial thalamus volume |
| Caudate volume (left and right)* | Medial-geniculate thalamus volume |
| Thalamus volume (left right)* | Mediodorsal thalamus volume |
| Pallidum volume (left and right)* | Mammillothalamic tract (MTT) thalamus volume |
| Putamen volume (left and right)* | Posterior thalamus volume |
| Amygdala volume (left and right)* | Pulvinar thalamus volume |
| Hippocampus volume (left and right)* |  |
| Claustrum volume |  |
| Internal pallidum volume |  |
| External pallidum volume |  |

*including left-hemispheric and right hemispheric volumes

**Supplementary Table 2**. Selected Methylation Probes for Computing Methylation Profile Score for BD

| Probe | Chromosome | Pos | Strand | Island | UCSC Gene |
| --- | --- | --- | --- | --- | --- |
| cg00111893 | chr6 | 109103695 | - | OpenSea | ZNF259P1 |
| cg20803857 | chr2 | 107502615 | + | Island | ST6GAL2 |
| cg08290675 | chr11 | 23425026 | + | N_Shore |  |
| cg05697637 | chr4 | 42659358 | - | Island | ATP8A1 |
| cg00922131 | chr2 | 38603784 | - | Island | ATL2 |
| cg14950747 | chr19 | 3821095 | + | Island | ZFR2 |
| cg04190888 | chr10 | 49879914 | + | OpenSea |  |
| cg02064336 | chr3 | 133167025 | + | OpenSea | BFSP2 |
| cg04602414 | chr1 | 35928126 | - | OpenSea | KIAA0319L |
| cg15504909 | chr14 | 65708285 | - | OpenSea |  |
| cg25223140 | chr7 | 24376318 | + | OpenSea |  |
| cg13043748 | chr20 | 431081 | + | OpenSea | TBC1D20 |
| cg01215538 | chr20 | 4927568 | - | OpenSea | SLC23A2 |
| cg10669351 | chr3 | 24007477 | - | OpenSea | NR1D2 |
| cg01062126 | chr16 | 104539 | + | S_Shore | SNRNP25 |
| cg03030879 | chr14 | 75389066 | - | N_Shore | RPS6KL1 |
| cg17733649 | chr7 | 151387983 | - | OpenSea | PRKAG2 |
| cg26568189 | chr1 | 44330548 | + | OpenSea | ST3GAL3 |
| cg16538953 | chr22 | 17089603 | + | N_Shore | TPTEP1 |
| cg08202972 | chr16 | 54851464 | + | OpenSea |  |
| cg04761450 | chr13 | 41172989 | - | OpenSea | FOXO1 |
| cg03274573 | chr7 | 101345806 | - | OpenSea |  |
| cg15997778 | chr7 | 116853801 | - | OpenSea | ST7 |
| cg17315967 | chr11 | 119038852 | - | N_Shore | NLRX1 |
| cg06654369 | chr7 | 158793845 | - | S_Shelf |  |
| cg26236440 | chr2 | 113341947 | - | Island | CHCHD5 |
| cg23851515 | chr2 | 51057218 | - | OpenSea | NRXN1 |
| cg12118239 | chr5 | 133286811 | - | OpenSea |  |
| cg15741931 | chr1 | 154192162 | + | N_Shore | UBAP2 |
| cg22533560 | chr7 | 29028662 | + | OpenSea | LOC100506497 |
| cg05863094 | chr1 | 153610491 | - | S_Shelf | CHTOP |
| cg03888000 | chr8 | 41444559 | - | OpenSea | AGPAT6 |
| cg24272313 | chr16 | 11021922 | - | OpenSea |  |
| cg27394486 | chr15 | 24921054 | - | Island | C15orf2 |
| cg09065742 | chr19 | 7692363 | + | N_Shore | XAB2 |
| cg11626857 | chr8 | 81615262 | + | OpenSea | ZNF704 |

**Supplementary Table 3**. Significant SNP-CpG Pairs from me-QTL Analyses. All SNPs are significantly (FDR<0.05) associated with the probe the cg00111893 (chr6:109103695, gene: ZNF259P1).

| SNP | Position | Allele | Gene |
| --- | --- | --- | --- |
| rs75838716 | 145445166 | G/A |  |
| rs12522369 | 155689310 | C/T |  |
| rs114044304 | 86569804 | C/T | CLCA4 |
| rs76419373 | 104462050 | G/A |  |
| rs17234823 | 6809820 | G/A | GRM7 |
| rs112771197 | 98863705 | A/G |  |
| rs183386238 | 67679846 | G/A | CPA6 |
| rs79474224 | 14082677 | T/G | NFIB |
| rs117340337 | 8802866 | C/A/T |  |
| rs117487916 | 7780765 | G/A | DNAH2 |
| rs6069771 | 56540778 | A/G/T |  |
| rs61934298 | 93402390 | C/A/T | NUDT4 |
| rs147953648 | 64345933 | G/A | CSNK1G1 |
