# Supplementary figures and images for: Genetic and environment effects on structural neuroimaging endophenotype for bipolar disorder: a novel molecular approach
Source: Transl Psychiatry. 2022 Apr 4;12:137. doi: 10.1038/s41398-022-01892-3 (PMC8980067; doi:10.1038/s41398-022-01892-3)

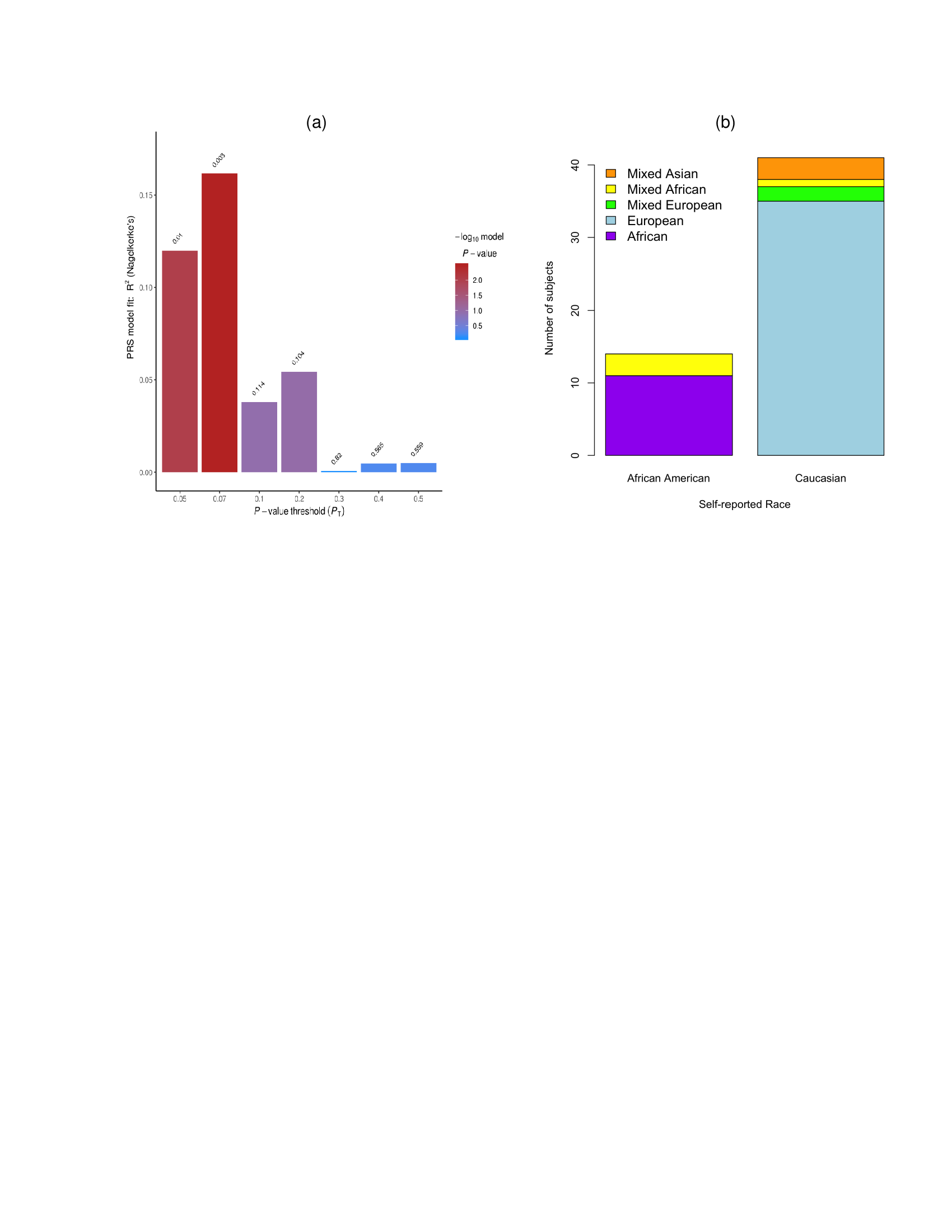

Supplement: Supplementary file 2 — Supplementary Figure 1 [file 41398_2022_1892_MOESM2_ESM.tif]

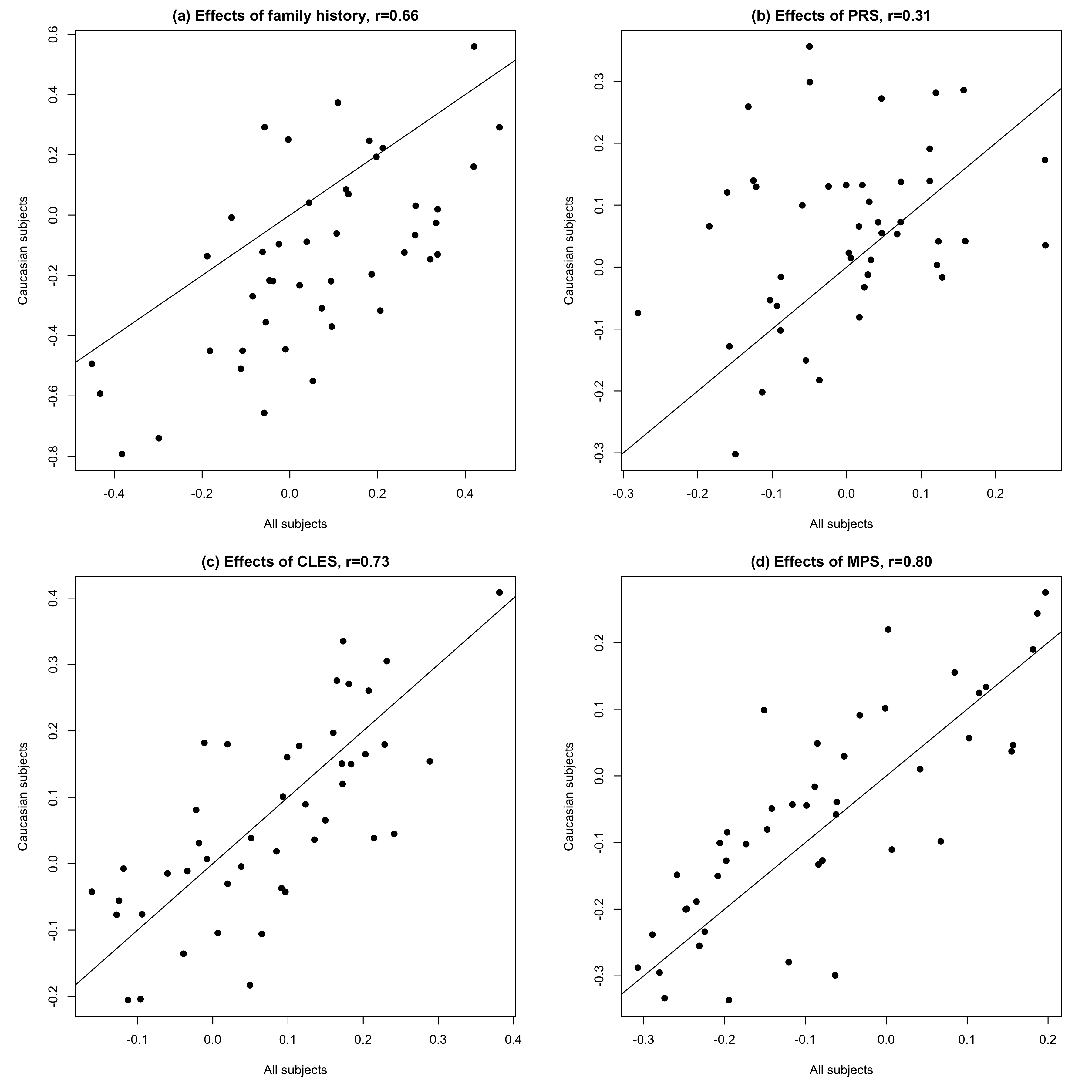

Supplement: Supplementary file 3 — Supplementary Figure 2 [file 41398_2022_1892_MOESM3_ESM.tif]
